# Association of serum albumin levels and stroke risk in adults over 40 years: A population-based study

**DOI:** 10.1097/MD.0000000000034848

**Published:** 2023-09-08

**Authors:** Yu Wang, Yangping Zhuang, Hanlin Huang, Jun Ke, Shirong Lin, Feng Chen

**Affiliations:** a Shengli Clinical Medical College of Fujian Medical University, Fuzhou City, China; b Department of Emergency, Fujian Provincial Hospital, Fuzhou City, China; c Fujian Key Laboratory of Emergency Medicine, Fujian Provincial Hospital, Fuzhou City, China; d Fujian Provincial Institute of Emergency Medicine, Fuzhou City, China; e Fujian Emergency Medical Center, Fuzhou City, China.

**Keywords:** cross-sectional study, NHANES, population-based study, serum albumin levels, stroke risk

## Abstract

This study assessed the relationship between serum albumin levels and adult stroke risk. From the 2009 to 2018 National Health and Nutrition Examination Survey, we performed a cross-sectional study with 17,303 participants who were 40 years of age or higher. A multivariate logistic regression model investigated serum albumin levels and stroke. To investigate apparent nonlinear connections, smoothed curve fitting was used. When a nonlinear relationship was discovered, the inflection point was determined using a recursive method. Serum albumin levels were significantly and inversely linked with the risk of stroke after controlling for possible variables [odds ratio 0.02, 95% confidence interval (0.00, 0.18), *P* = .0003]. An examination of subgroups revealed that the inverse relationship between serum albumin levels and risk of stroke was statistically significant in men, participants under 60 years old, non-diabetic participants, and hypertensive participants. Serum albumin levels and the risk of stroke were negatively correlated. An increased risk of stroke was linked to lower serum albumin levels.

## 1. Introduction

With 16.8% of all fatalities caused by cerebrovascular illness, it is one of the leading causes of mortality globally.^[1]^ Stroke is the most prevalent kind of cerebrovascular illness. A stroke, which can arise from cerebral infarction or intracranial bleeding, is a neurological dysfunction brought on by abrupt localized injury to the nervous system in the brain.^[2,3]^ As a result of cerebral venous sinus thrombosis can produce vascular occlusion, severe headaches, convulsions, elevated corpus callosum, and bleeding in the subarachnoid space from blood filling up to the infarct stroke may also result in neurological impairments.^[4,5]^ Stroke is a dangerous illness that jeopardizes public health and has a low occurrence rate. Hence, identifying the risk factors for stroke is crucial from a clinical standpoint.^[6–10]^

In serum, albumin is a common, non-glycosylated, and highly adaptable transport protein.^[11]^ It regulates plasma colloid osmotic pressure, transports numerous compounds in plasma, and serves as one of the crucial biochemical markers for treatments.^[12,13]^ Albumin also possesses actions that are anti-inflammatory, antioxidant, and anti-platelet coagulation.^[14–17]^ These factors might affect how a stroke starts and develops. While it has received more attention, the connection between albumin levels and the risk of stroke has not yet been thoroughly understood.^[18]^

Serum albumin is crucial in determining the risk of stroke. On the one side, this association may reveal a novel component linked to stroke pathophysiology and offer insight into how albumin may play a role in this process. On the other hand, it suggests a course for future clinical and basic medical study. Also, this research may offer a preliminary, scientific, proof medical evaluation of whether variations in serum albumin levels impact stroke frequency. We investigated the link between serum albumin levels and stroke risk in persons above 40 years of age to see if there is any correlation between them using a cross-sectional analysis of a nationally representative National Health and Nutrition Examination Survey-based dataset.

## 2. Materials and methods

### 2.1. Data and example sources

The National Center for Health Statistics, which gathers data on possible health risk factors and nutritional status of the noninstitutionalized U.S. population, provided the NHANES database, a nationwide demographically cross-sectional survey. A sophisticated stratified, multi-stage random group sampling design was created to get a representative sample of the total American population. To examine their physical and psychological state, participants had a standard questionnaire at home, a health screening at a mobile screening center, and laboratory analysis to get their laboratory results.^[19]^ The link between serum albumin levels and stroke risk was evaluated using data from 5 NHANES periods from 2009 to 2018. It kept participants older than 40 years with whole serum albumin levels and stroke data.

### 2.2. Ethics statement

The National Center for Health Statistics Research Ethics Review Committee approved the NHANES study procedures. All survey respondents provided signed informed permission, and poll respondents under 16 provided approval from their parents or legal guardians. Accessible information on the NHANES original study design and data may be found at https://www.cdc.gov/nchs/nhanes/.

### 2.3. Serum albumin levels and stroke status definition

Participants in the NHANES provide biological samples, which are collected at a standardized mobile screening facility. A 2-color digital endpoint technique, the DcX800 method, is employed to gauge serum albumin content. The combination is created when albumin and the Bromcresol Purple reagent interact. The system keeps track of any variations in absorbance at 600 nm. The amount of albumin in the sample is inversely correlated with the change in absorbance. Our research considered serum albumin levels (g/dL) as an exposure variable.

We used these data to establish stroke status by integrating identity medical diagnoses and a standardized medical condition questionnaire filled out during individual interviews. A skilled interviewer asked participants, “Has a doctor or other health professional ever told you that you had a stroke?” They gave a yes/no response. They were regarded as unavailable if they could not respond or did not know.

### 2.4. Covariates

Likewise, covariates such as gender (male/female), age (year), race (Mexican American/other Hispanic/non-Hispanic White/non-Hispanic Black/other races), an education level (high school and below/above high school), smoking (yes/no), alcohol use status (drinks), body mass index (BMI, kg/m^2^), hypertension (yes/no), diabetes (yes/no), serum creatinine (SCr, µmol/L), blood urea nitrogen (BUN, mmol/L), serum uric acid (UA, µmol/L), glucose (mmol/L), white blood cells (WBC, 1000 cells/uL), triglyceride (TG, mmol/L), high-density lipoprotein-cholesterol (HDL-C, mmol/L) and low-density lipoprotein-cholesterol (LDL-C, mmol/L) were taken into account in our research. Participants of ages, genders, races, levels of education, smoking, alcohol use status, hypertension, and diabetes were all asked about using the Computer-Assisted Personal Interviewing system at home by trained observers. The following questions were asked of participants to determine their status concerning smoking, alcohol usage, blood pressure, and diabetes: “Do you now smoke cigarettes?,” “In the past 12 months, on those days that you drank alcoholic beverages, on average, how many drinks did you have?,” “Have you ever been told by a doctor or other health professional that you had hypertension, also called high blood pressure?,” “Other than during pregnancy, have you ever been told by a doctor or health professional that you have diabetes or sugar diabetes?” Participants gave a yes or no response. They were classified as missing information if they declined to respond or did not have the information. By calculating the participants for height and weight, the BMI was determined. When the Beckman UniCel DxC800 Synchron simultaneous kinetic rate technique was employed and based on biochemical analysis, the LDL-C, HDL-C, TG, SCr, BUN, UA and glucose levels were determined. The Beckman Coulter counting and calibrating method is utilized to detect leukocytes, and a computerized dilution and mixing unit is used to handle the sample. The complete measurement methods for these variables are all accessible to the public at www.cdc.gov/nchs/nhanes/.

### 2.5. Statistical analysis

The Centers for Disease Control and Prevention recommended that all statistical tests be carried out using the proper NHANES sampling weights and accounting for detailed multi-stage subgroup surveys. Means and standard deviations portray continuous variables, whereas percentages describe categorical variables. The individual characteristics were compared using a weighted Student’s *t* test for continuous data or a weighted chi-square test for categorical variables. In 3 distinct models, the relationship between serum albumin levels and the risk of stroke was examined using multivariate logistic regression. Covariate adjustments were not made in Model 1 at all. Age, gender and race can all be adjusted in Model 2. Age, sex, race, education level, hypertension, diabetes, BMI, SCr, BUN, UA, WBC, HDL-C, LDL-C, TG, glucose, smoking and alcohol use status were all considered when creating Model 3. It should be mentioned that owing to a substantial departure from the normally distributed, serum albumin levels were represented by the ln transform while regression analysis was conducted. Sex (male/female), age (<60/≥60 years), smoking (yes/no), alcohol use status (≤3/>3 drinks), hypertension (yes/no) and diabetes (yes/no) were used as classification variables in an investigation of the relationship between serum albumin levels and stroke risk. Furthermore, these classification characteristics were considered as pre-determined possible impact modifiers. We introduced an interaction term to check the heterogeneity of correlations across subgroups. In addition, the nonlinear link between serum albumin levels and stroke risk was examined using generalized additivity models and smoothed curve fitting. A recursive method was created to determine the inflection point of the connection between serum albumin levels and stroke risk when nonlinearity was found. The impact value was calculated using a double-segmented logistic regression model on both sides of the inflection point. Missing values for categorical variables were entered using the distribution of the available instances while missing values for continuous variables were inserted using the median. R version 3.4.3 and Empower software (www.empowerstats.com) were used for all analyses; *P* < .05 was considered statistically significant.

## 3. Results

### 3.1. Participant selection and baseline characteristics

**Figure 1.**
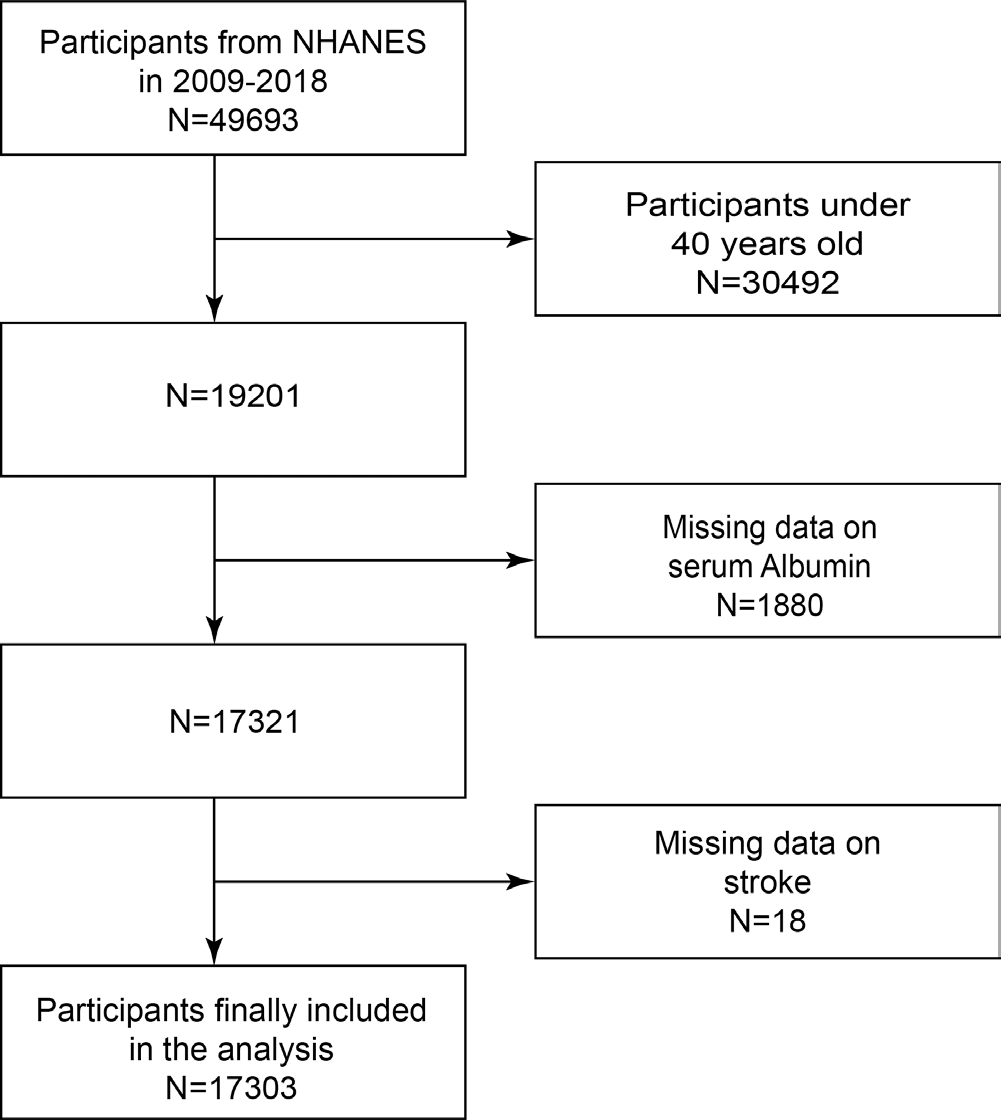
The participant inclusion procedure for the research.

Forty-nine thousand six hundred ninety-three individuals participated in the 5 survey cycles from 2009 to 2018. The remaining 17,303 participants were included in the research after those under 40, those without serum albumin levels and those without stroke data were phased out. Figure 1 depicts an example procedure.

For some 17,303 people enrolled in this study, Table 1 provides the baseline characteristics of the study population based on quartiles of serum albumin levels (Q1: 4 g/dL; Q2: 4–4.1 g/dL; Q3: 4.2–4.3 g/dL; and Q4: >4.3 g/dL). Among the 4 quartiles of serum albumin levels, there were statistically significant variations in age, sex, race, education level, smoking, alcohol use status, BMI, diabetes, hypertension, UA, SCr, BUN, glucose, WBC, HDL-C, LDL-C and TG (all *P* < .05). In Q1, Q2, Q3 and Q4, the incidence of stroke was 8.61%, 5.56%, 4.78%, and 3.88%, correspondingly. In this study, males, HDL-C, LDL-C, TG and alcohol use status were associated with higher serum albumin levels. In contrast, age, smoking, hypertension, diabetes, SCr, BUN, glucose, WBC and stroke patients were associated with lower serum albumin levels (all *P* < .05).

**Table 1:** Weighted characteristics of the study population based on serum albumin concentration quartile.

Variables	Serum albumin concentration, g/dL				*P* value
	Quartile 1 (n = 3892)	Quartile 2 (n = 3848)	Quartile 3 (n = 4329)	Quartile 4 (n = 5234)	
Age (yr)	61.42 ± 12.59	60.49 ± 12.18	59.53 ± 11.91	57.88 ± 11.75	<.001
Gender, % (SE)					
Male	37.10	43.53	50.80	59.11	<.001
Female	62.90	56.47	49.20	40.89	
Race, % (SE)					
Mexican American	13.05	14.40	15.43	13.35	<.001
Other Hispanic	9.82	10.55	10.70	10.83	
Non-Hispanic White	36.77	40.77	42.09	44.96	
Non-Hispanic Black	30.47	22.90	18.69	15.11	
Other Races	9.89	11.38	13.10	15.74	
Education level, % (SE)					
High school and below	29.01	26.67	26.57	23.38	<.001
Above high school	70.99	73.33	73.43	76.62	
Smoking, % (SE)					
Yes	40.10	39.50	37.05	36.28	.030
No	59.90	60.50	62.95	63.72	
BMI, % (SE)					
˂25 kg/m^2^ normal	17.92	20.57	25.71	31.84	<.001
25–30 kg/m^2^ overweight	26.45	33.29	37.63	40.73	
˃30 kg/m^2^ obese	55.63	46.14	36.67	27.43	
Hypertension, % (SE)					
Yes	55.78	50.77	46.55	43.14	<.001
No	44.22	49.23	53.45	56.86	
Diabetes, % (SE)					
Yes	26.32	20.45	16.57	14.51	<.001
No	70.08	76.27	79.80	82.39	
Borderline	3.60	3.27	3.63	3.10	
UA (µmol/L)	331.42 ± 94.96	327.55 ± 88.64	326.89 ± 83.75	332.15 ± 83.27	.006
SCr (µmol/L)	90.45 ± 73.80	82.50 ± 40.37	81.22 ± 42.08	80.70 ± 25.69	<.001
BUN (mmol/L)	5.75 ± 3.14	5.41 ± 2.36	5.29 ± 2.02	5.20 ± 1.94	<.001
Glucose (mmol/L)	6.51 ± 3.21	6.15 ± 2.54	5.95 ± 2.19	5.78 ± 1.90	<.001
WBC (1000 cells/µL)	7.41 ± 2.78	7.27 ± 6.94	7.05 ± 2.63	6.98 ± 2.24	<.001
HDL-C (mmol/L)	1.36 ± 0.41	1.37 ± 0.40	1.38 ± 0.42	1.43 ± 0.47	<.001
LDL-C (mmol/L)	2.83 ± 0.93	2.94 ± 0.92	3.02 ± 0.94	3.09 ± 0.96	<.001
TG (mmol/L)	1.39 ± 1.67	1.40 ± 1.11	1.44 ± 1.05	1.51 ± 1.09	.008
Alcohol use status (drinks)	2.27 ± 1.97	2.29 ± 2.13	2.39 ± 2.12	2.54 ± 2.27	<.001
Stroke, % (SE)					
Yes	8.61	5.56	4.78	3.88	<.001
No	91.39	94.44	95.22	96.12	

BMI = body mass index, BUN = blood urea nitrogen, SCr = serum creatinine, HDL-C = high density lipoprotein cholesterol, LDL-C = low density lipoprotein cholesterol, TG = triglycerides, UA = urea acid, WBC = white blood cell.

### 3.2. Assessment of the association between serum albumin levels and stroke

As indicated in Table 2, 3 multivariate logistic regression models were run to evaluate the relationship between serum albumin levels (ln transform) and stroke. Continuous serum albumin levels (ln transform) were shown to be inversely correlated with the risk of stroke in the crude model [odds ratio (OR) 0.02, 95% confidence interval (CI) (0.01, 0.04), *P* < .0001]. This inverse relationship remained high both after complete adjustment [OR 0.02, 95% CI (0.00, 0.18), *P* = .0003] and minimum adjustment [OR 0.04, 95% CI (0.02, 0.09), *P* < .0001] for relevant mediators. Individuals inside the Q4 group had a statistically significant 45% reduced risk of stroke compared to individuals in the Q1 group as a reference [OR 0.55, 95% CI (0.31, 0.98), *P* = .0414] when serum albumin was transformed to a categorical variable. When individuals in the Q2 and Q3 groups were linked to the reference in the Q1 group, this connection did not reach statistical significance [Q2: OR 0.98, 95% CI (0.59, 1.63), *P* = .9515; Q3: OR 0.65, 95% CI (0.37, 1.15), *P* = .1406]. The trend *P*, meanwhile, was likewise significant in the unadjusted model (trend *P* = .0319), the minimally adjusted model (trend *P* = .0001), and the wholly corrected model (trend *P* = .0000), according to sensitivity analysis.

**Table 2:** Associations between serum albumin concentration and stroke.

	OR (95% CI), *P* value		
	Crude model (Model 1)	Minimally adjusted model (Model 2)	Fully adjusted model (Model 3)
Serum albumin	0.02 (0.01, 0.04), <.0001	0.04 (0.02, 0.09), <.0001	0.02 (0.00, 0.18), 0.0003
Serum albumin (quartile)			
Quartile 1	Reference	Reference	Reference
Quartile 2	0.63 (0.52, 0.75), <.0001	0.67 (0.56, 0.81), <.0001	0.98 (0.59, 1.63), .9515
Quartile 3	0.53 (0.45, 0.64), <.0001	0.61 (0.51, 0.74), <.0001	0.65 (0.37, 1.15), .1406
Quartile 4	0.43 (0.36, 0.51), <.0001	0.54 (0.45, 0.66) <.0001	0.55 (0.31, 0.98), .0414
*P* for trend	<.0001	<.0001	.0319

Serum albumin (in g/dL) was ln-transferred because of a skewed distribution. Minimally adjusted for age, gender, race. Fully adjusted for age, gender, race, education level, smoking, BMI, hypertension, diabetes, alcohol use status, Cr, BUN, UA, glucose, WBC, HDL-C, LDL-C, and TG.

BMI = body mass index, BUN = blood urea nitrogen, CI = confidence interval, SCr = serum creatinine, HDL-C = high density lipoprotein cholesterol, LDL-C = low density lipoprotein cholesterol, OR = odds ratio, TG = triglycerides, UA = urea acid, WBC = white blood cell.

In the fully adjusted model, stroke risks for age, race, education level, smoking, hypertension, SCr and serum albumin levels (ln transform) remained strongly correlated (Table 3). Compared to Mexican Americans, non-Hispanic blacks had a 1.09-fold higher risk of stroke (*P* = .0267). Nonsmokers had a 49% lower risk of stroke (*P* = .0045) than smokers. Compared to hypertensives, non-hypertensives had a 51% higher risk of stroke (*P* = .0026). The risk of stroke increased by 1% for every unit higher SCr (*P* = .0077).

**Table 3:** Multivariate analysis of associations between various variables and stroke.

Variable	OR (95% CI)	*P* value
ln (serum albumin)	0.03 (0.00, 0.26)	.0014
Age (yr)	1.04 (1.01, 1.06)	.0020
Gender, % (SE)		
Male	Reference	.7328
Female	1.09 (0.68, 1.74)	
Race, % (SE)		
Mexican American	Reference	
Other Hispanic	1.38 (0.41, 4.71)	.6024
Non-Hispanic White	2.43 (0.92, 6.39)	.0728
Non-Hispanic Black	3.09 (1.14, 8.38)	.0267
Other races	2.63 (0.83, 8.36)	.1005
Education level, % (SE)		
High school and below	Reference	
Above high school	0.44 (0.29, 0.67)	.0001
Smoking, % (SE)		
Yes	Reference	
No	0.51 (0.32, 0.81)	.0045
BMI, % (SE)		
˂25 kg/m^2^ normal	Reference	
25–30 kg/m^2^ overweight	0.96 (0.56, 1.64)	.8880
˃30 kg/m^2^ obese	1.00 (0.57, 1.74)	.9864
Hypertension, % (SE)		
Yes	Reference	
No	0.49 (0.31, 0.78)	.0026
Diabetes, % (SE)		
Yes	Reference	
No	0.71 (0.40, 1.25)	.2340
Borderline	1.06 (0.42, 2.70)	.9038
Alcohol use status (drinks)	0.95 (0.86, 1.06)	.3430
SCr (µmol/L)	1.01 (1.00, 1.01)	.0077
BUN (mmol/L)	0.98 (0.88, 1.08)	.6585
UA (µmol/L)	1.00 (1.00, 1.00)	.7817
Glucose (mmol/L)	0.97 (0.87, 1.09)	.6369
WBC (1000 cells/uL)	1.00 (0.92, 1.08)	.9444
HDL-C (mmol/L)	0.92 (0.56, 1.53)	.7535
LDL-C (mmol/L)	0.94 (0.75, 1.19)	.6215
TG (mmol/L)	0.90 (0.64, 1.25)	.5240

BMI = body mass index, BUN = blood urea nitrogen, CI = confidence interval, SCr = serum creatinine, HDL-C = high density lipoprotein cholesterol, LDL-C = low density lipoprotein cholesterol, OR = odds ratio, TG = triglycerides, UA = urea acid, WBC = white blood cell.

Table 4 shows the results of subgroup analysis by gender, age, alcohol use status, smoking, diabetes and hypertension. Men [OR 0.01, 95% CI (0.00, 0.21)], participants 60 years of age and younger [OR 0.00, 95% CI (0.00, 0.09)], participants without diabetes [OR 0.02, 95% CI (0.00, 0.28)], participants with hypertension [OR 0.03, 95% CI (0.00, 0.34)], participants who drank at least 3 drinks [OR 0.02, 95% CI (0.00, 0.24)], and participants who did not smoke [smokers: OR 0.02, 95% CI (0.00, 0.48); nonsmokers: OR 0.04, 95% CI (0.00,0.99)] all had an inverse relationship between serum albumin levels and risk of stroke.

**Table 4:** Relationships of the serum albumin level (ln transformation) with stroke in subgroups.

Subgroup	Adjusted OR (95% CI)	*P* for interaction
Gender		
Males	0.01 (0.00, 0.21)	.3713
Females	0.11 (0.00, 4.34)	
Age (yr)		
≤60	0.00 (0.00, 0.09)	.0210
˃60	0.78 (0.03, 20.64)	
Smoking		
Yes	0.02 (0.00, 0.48)	.8437
No	0.04 (0.00, 0.99)	
Alcohol use status		
≤3	0.02 (0.00, 0.24)	.5944
˃3	0.10 (0.00, 30.51)	
Hypertension		
Yes	0.03 (0.00, 0.34)	.8710
No	0.04 (0.00, 3.61)	
Diabetes		
Yes	0.05 (0.00, 2.44)	.7174
No	0.02 (0.00, 0.28)	

Adjusted for age, gender, race, education level, smoking, BMI, hypertension, diabetes, alcohol use status, Cr, BUN, UA, glucose, WBC, HDL-C, LDL-C, and TG.

BMI = body mass index, BUN = blood urea nitrogen, CI = confidence interval, SCr = serum creatinine, HDL-C = high density lipoprotein cholesterol, LDL-C = low density lipoprotein cholesterol, OR = odds ratio, TG = triglycerides, UA = urea acid, WBC = white blood cell.

**Figure 2.**
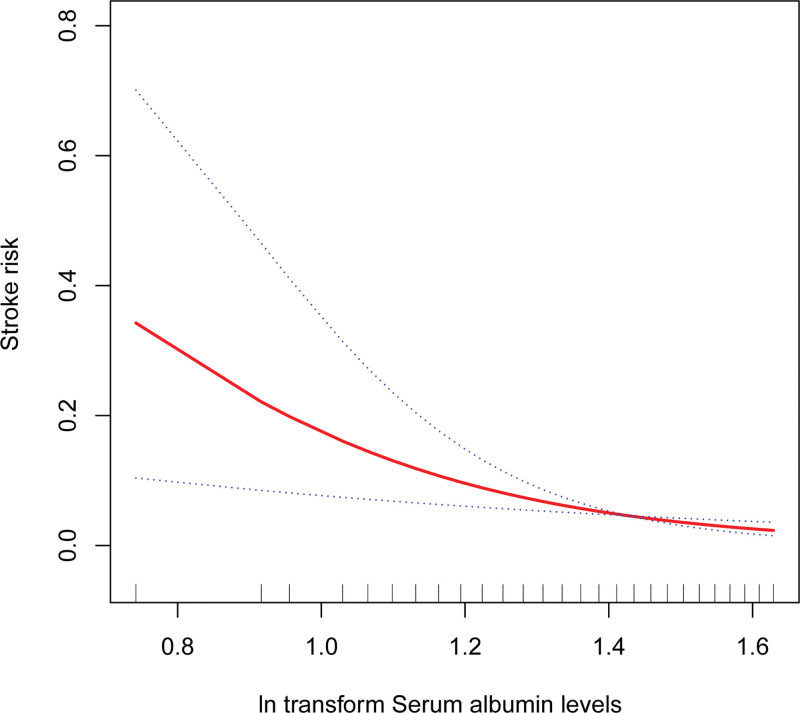
Smooth curve fitting. The relationship between serum albumin levels and stroke risk. The blue curve represents the 95% confidence interval.

An inverse relationship between serum albumin levels and stroke risk was also revealed by smoothing the curve fit (Fig. 2). It appears to have a threshold impact with a breakpoint of 1.48. However, this link was not statistically significant (log-likelihood ratio 0.311, Table 5).

**Table 5:** Threshold effect.

Outcome	Stroke risk
Model I	
A straight-line effect	0.03 (0.00, 0.28)
Model II	
Fold points (K)	1.48
<K-segment effect 1	0.06 (0.00, 0.73)
>K-segment effect 2	0.00 (0.00, 77.94)
Effect size difference of 2 vs. 1	0.00 (0.00, 3537.02)
Equation predicted values at break points	−3.27 (−3.57, −2.97)
Log likelihood ratio tests	0.311

Result variable: stroke.

Exposure variables: serum albumi.

Results are expressed as OR (95% CI).

Adjusted for age, gender, race, education level, smoking, BMI, hypertension, diabetes, alcohol use status, Cr, BUN, UA, glucose, WBC, HDL-C, LDL-C, and TG.

BMI = body mass index, BUN = blood urea nitrogen, CI = confidence interval, SCr = serum creatinine, HDL-C = high density lipoprotein cholesterol, LDL-C = low density lipoprotein cholesterol, OR = odds ratio, TG = triglycerides, UA = urea acid, WBC = white blood cell.

**Figure 3.**
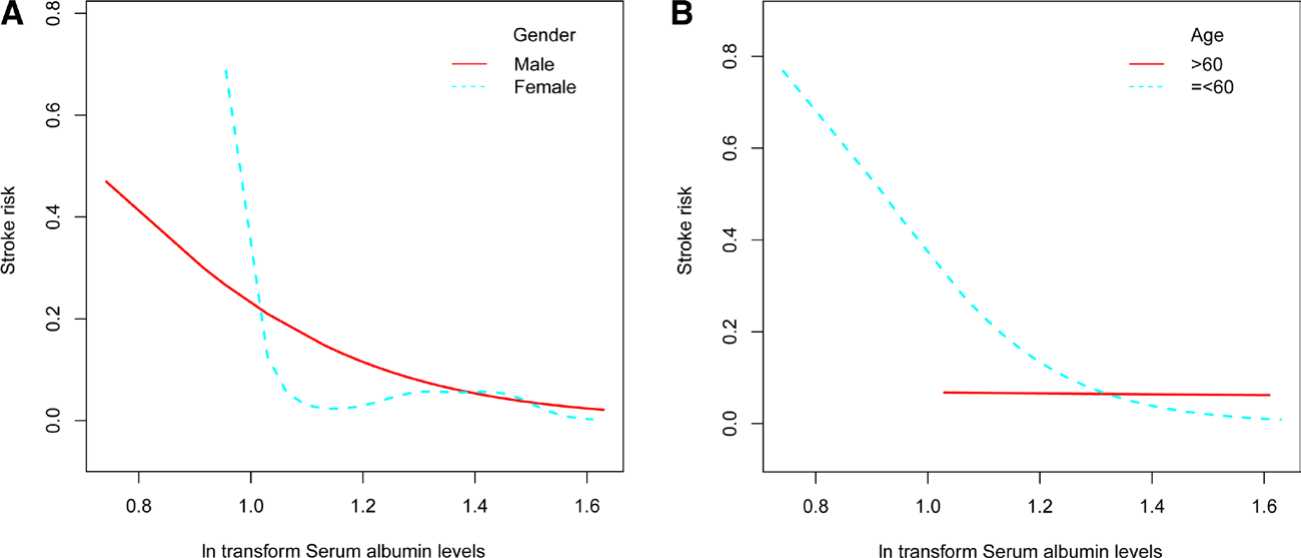
The relationship between serum albumin levels and stroke risk hierarchical by age and gender. Adjust for: age, gender, race, education level, smoking, BMI, hypertension, diabetes, alcohol use status, Cr, BUN, UA, glucose, WBC, HDL-C, LDL-C, TG. When gender or age was the hierarchical variable, it were not adjusted. BMI = body mass index, BUN = blood urea nitrogen, SCr = serum creatinine, HDL-C = high density lipoprotein cholesterol, LDL-C = low density lipoprotein cholesterol, TG = triglycerides, UA = urea acid, WBC = white blood cell.

**Figure 4.**
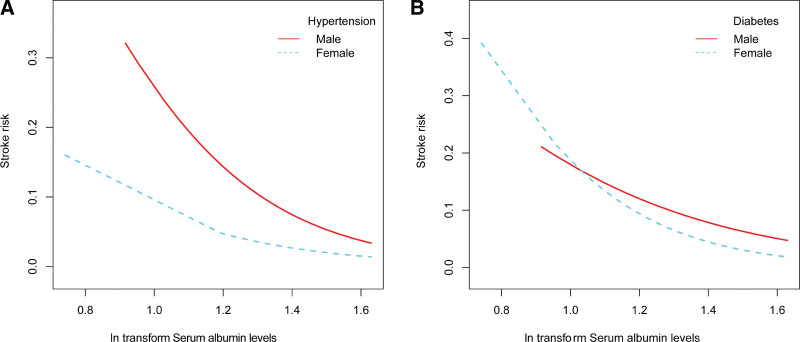
The relationship between serum albumin levels and stroke risk hierarchical by hypertension and diabetes. Adjust for: age, gender, race, education level, smoking, BMI, hypertension, diabetes, alcohol use status, Cr, BUN, UA, glucose, WBC, HDL-C, LDL-C, TG. When hypertension and diabetes was the hierarchical variable, it were not adjusted. BMI = body mass index, BUN = blood urea nitrogen, SCr = serum creatinine, HDL-C = high density lipoprotein cholesterol, LDL-C = low density lipoprotein cholesterol, TG = triglycerides, UA = urea acid, WBC = white blood cell.

**Figure 5.**
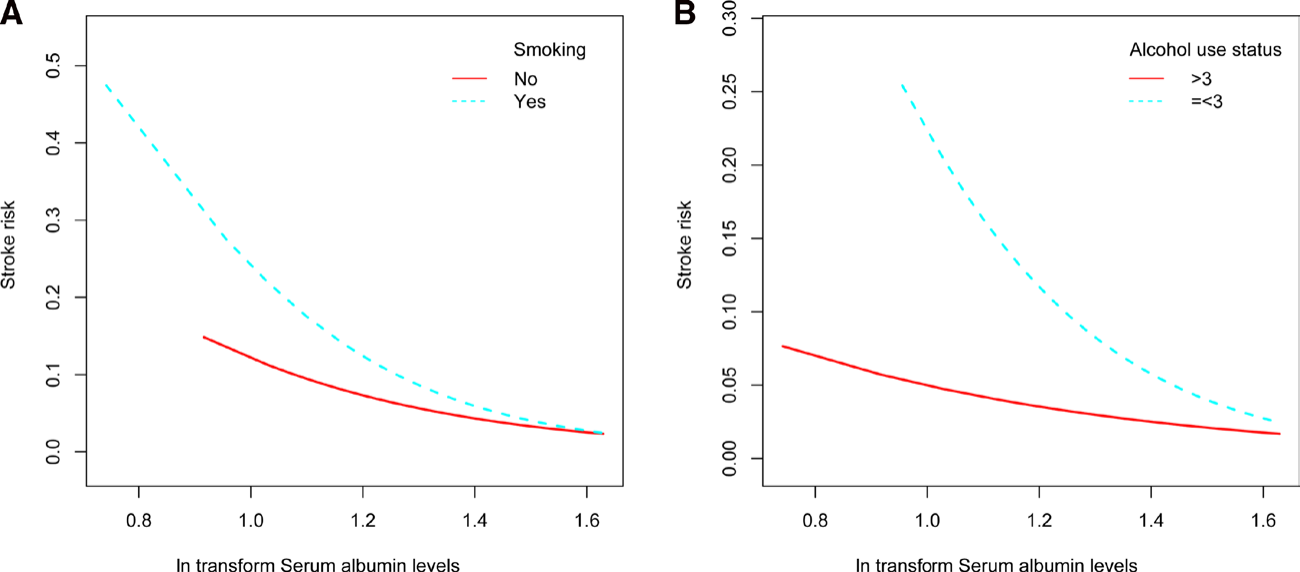
The relationship between serum albumin levels and stroke risk hierarchical by smoking and alcohol use status. Adjust for: age, gender, race, education level, smoking, BMI, hypertension, diabetes, alcohol use status, Cr, BUN, UA, glucose, WBC, HDL-C, LDL-C, TG. When smoking and alcohol use status was the hierarchical variable, it were not adjusted. BMI = body mass index, BUN = blood urea nitrogen, SCr = serum creatinine, HDL-C = high density lipoprotein cholesterol, LDL-C = low density lipoprotein cholesterol, TG = triglycerides, UA = urea acid, WBC = white blood cell.

Hierarchical smooth curve fitting revealed an L-shaped relationship between serum albumin levels and stroke risk in women (Fig. 3A), a weakened relationship in participants over 60 years (Fig. 3B), a roughly negative relationship independent of hypertension and diabetes status (Fig. 4A and B), and a roughly negative relationship independent of smoking and alcohol use status (Fig. 5A and B).

## 4. Discussion

Data from the NHANES 2009-2018 database were examined for this investigation. Serum albumin levels were inversely correlated with the risk of stroke in individuals over 40 after controlling for variables using multiple logistic regression, and smoothed curve fitting revealed the same findings.

Stroke is a dangerous illness that puts the lives of people in peril. Identifying the risk factors for Stroke has been a widespread study issue. Serum albumin levels with the onset and course of stroke have already been examined in several investigations. A cohort of studies with 24 years of follow-up discovered that low blood albumin levels were linked to a higher risk of total and ischemic stroke. The result agrees with what we found.^[20]^ Also, they conducted a stratified analysis and discovered multivariate [HR, 95% CI] of [1.44, (1.07, 1.92)], [1.48, (1.03, 2.11)], and [1.63, (1.11, 2.39)], separately, for total and ischemic stroke in the lowest versus highest quartile of serum albumin in men and women, respectively. Similar inverse relationships were found for each ischemic stroke subtype.^[20]^ Nevertheless, after fitting a smoothed curve stratified by sex, we discovered a negative correlation between serum albumin and stroke risk in males and an L-shaped connection in women. Due to the impact of controlling for too many confounders in a small sample size, this disparity may have occurred. Høstmark et al^[21]^ found that decreased blood albumin was related to an increased risk of self-reported stroke in a cross-sectional analysis of the Oslo Health Study in Norway. Babu et al^[22]^ found that unfavorable outcomes were decreased in acute stroke patients with relatively high serum albumin levels. According to a meta-analysis of data from the third Chinese National Stroke Registry, the prognosis was consistently worse over the 3-month follow-up for every 10 g/L decrease in blood albumin [adjusted OR 1.17, 95% CI (1.01, 1.35); adjusted HR 1.86, 95% CI (1.30, 2.64)]. According to a meta-analysis of data from the third Chinese National Stroke Registry, throughout the 3-month follow-up, the relationship between every 10 g/L decrease in blood albumin and the prognosis was consistently reversed [adjusted OR 1.17, 95% CI (1.01, 1.35); adjusted HR 1.86, 95% CI (1.30, 2.64)]. In the meta-analysis, for each 1 g/L reduction, adverse functional outcome was pooled for 3 studies: [OR 1.03, 95% CI (1.02, 1.05)] and mortality for 5 studies: [HR 1.07, 95% CI (1.03, 1.11)]. Low serum albumin levels predict poor outcomes in individuals with acute ischemic stroke or transient ischemic attack.^[23]^ Serum albumin levels are also related to acute ischemic stroke, a severe stroke of therapeutic importance. However, the definition of stroke in our study was entirely open-ended. As a result, an analysis of acute ischemic stroke subgroups was impossible.

Serum albumin levels and the risk of stroke, including acute ischemic stroke and transient ischemic attack, have also been linked in several geographical populations, according to other research.^[24,25]^ In a prospective cohort study of 2986 individuals in the Northern Manhattan population with a 12-year follow-up, it was found that compared with those with levels of 4.6 to 5.5 g/dL (Tertile3, reference), participants with serum albumin levels of 2.7 to 4.2 g/dL (Tertile1) had all strokes [HR 1.76, 95% CI (1.32, 2.35)], ischemic stroke [HR 1.67, 95% CI (1.21, 2.29)], myocardial infarction stroke [HR 1.92, 95% CI (1.10, 3.34)], and cryptogenic stroke [HR 2.59, 95% CI (1.21, 5.53)]. Low blood albumin levels have been linked to ischemic stroke, particularly myocardial infarction and cryptogenic subtype.^[25]^ Idicula et al^[24]^ found that high serum albumin was related to improved prognosis and decreased mortality in individuals with ischemic stroke. Babu et al^[22]^ found that patients with acute ischemic stroke have a higher risk of recurrence when their blood albumin levels are lower. Both our research results and all those mentioned above could imply that serum albumin is inversely associated with stroke risk and that high levels of serum albumin may be a protective factor against stroke, even though the covariate factors modified for in the study were also not the same and the significance of the present evaluation varied. The findings have been confirmed in several locations and by various study teams. After stratified analysis, our study discovered an inverse relationship between serum albumin levels and the risk of stroke in men [OR 0.01, 95% CI (0.00, 0.21)], participants 60 years of age and younger [OR 0.00, 95% CI (0.00, 0.09)], participants without diabetes [OR 0.02, 95% CI (0.00, 0.28)], participants with hypertension [OR 0.03, 95% CI (0.00, 0.34)], participants who drank at least 3 drinks [OR 0.02, 95% CI (0.00, 0.24)], and participants who did not smoke [smokers: OR 0.02, 95% CI (0.00, 0.48); nonsmokers: OR 0.04, 95% CI (0.00, 0.99)].

It has been suggested that the anti-inflammatory, antioxidant, and anti-platelet accumulation properties of serum albumin may play a part in the influence of serum albumin on the pathophysiology of stroke.^[14,26,27]^ Serum albumin levels are a marker of the severity of inflammation; when immune cells such as macrophages are activated due to inflammation, more cytokines are produced, which causes the liver to switch from producing albumin to producing other acute phase proteins.^[28]^ One of the antioxidants in whole blood and one of the markers for serious to moderate anemia is serum albumin levels. Sulfhydryl groups, which comprise a large portion of the total nucleophiles in plasma and scavenge reactive oxygen and nitrogen species, are abundant in serum albumin. Nitric oxide and bilirubin are other molecules transported by serum albumin and offer further defense against oxidative stress.^[29]^ In addition to having an antioxidant function, albumin may interact with additional ions, including silver, iron, and zinc, to prevent the synthesis of reactive oxygen species.^[30,31]^ By blocking endotoxin-induced inflammation and oxidative stress pathways, 4% human serum albumin therapy was also beneficial against survivability and endothelium dysfunction in mice.^[32]^ By blocking histones in a charge-dependent way and limiting platelet aggregation by attaching to antithrombin 4, albumin also adversely influences platelet aggregation, which has a significant impact.^[33,34]^

The NHANES database was used to gather the data for our investigation. The latest survey advantages include accurate data, a sizable random sample from an ethnically varied population more typical of the U.S. population, and the absence of previous research with sizable sample sizes for comparable topics. Second, to lessen the potential impact of confounding factors on observational studies, we built multiple logistic regression models throughout data analysis by meticulously adjusting for various potential confounders and combining significant factors in diverse types as continuous and categorical variables. To get consistent findings in subgroups and depict the connection between the variables in an understandable fashion, we also did subgroup analysis, interaction analysis, and smoothed curve fitting.

Our study does, however, have certain drawbacks. First, because our research was cross-sectional, we can only draw confirmatory factors, not causal inferences. Consequently, based on our investigation, more prospective studies may be conducted. Therefore, additional prospective studies could be carried out based on our research. Second, evaluating serum albumin concentrations concerning the subtype and degree of stroke is constrained because individuals with stroke could not provide precise information about their stroke. Third, despite the work carried out in prior studies using NHANES data, it must be noted that there might be remember bias in addition to the absence of some essential cerebrovascular indicators because the outcome indicators in our study were obtained by standardized medical questionnaires rather than by collecting objective measurements. Fourth, the data used in our study were collected from middle-aged and older Americans, which to some degree, limits the generalizability of our conclusions to other nations and areas and calls for additional research.

Additionally, although we adjusted some covariates during the trial, a few covariates that were not of concern could still impact the result markers. Based on new results, future scholars can keep refining the design of experiments. We discovered an inverse relationship between blood albumin amounts and stroke risk. Further research is necessary to determine the fundamental process of this and the therapeutic application of serum albumin amounts to lower stroke risk.

## 5. Conclusions

Our investigation demonstrated a consistent inverse relationship between serum albumin levels and stroke risk. Higher expected future research will still be required to support or refute our findings.

## Acknowledgments

We appreciate the data coming from the National Health and Nutrition Examination Surveys.

## Author contributions

**Conceptualization:** Yu Wang, Feng Chen.

**Data curation:** Hanlin Huang, Jun Ke, Shirong Lin.

**Formal analysis:** Hanlin Huang, Jun Ke, Shirong Lin.

**Supervision:** Yangping Zhuang, Jun Ke.

**Writing – original draft:** Yu Wang, Yangping Zhuang, Jun Ke.

**Writing – review & editing:** Feng Chen.
